# ‘I Have my Beliefs, but Then I Have my Reality’: Reflections of Black and South Asian Parents Living in England on Screening and Genetic Diagnosis in Pregnancy

**DOI:** 10.1002/pd.6782

**Published:** 2025-03-20

**Authors:** Michelle Peter, Rashida Baptiste, Rachael Buabeng, Lily Islam, Jane Fisher, Kerry Leeson‐Beevers, Melissa Hill, Lyn S. Chitty

**Affiliations:** ^1^ North Thames Genomic Laboratory Hub Great Ormond Street Hospital for Children NHS Foundation Trust London UK; ^2^ Genetics and Genomic Medicine UCL Great Ormond Street Institute of Child Health London UK; ^3^ Mummy's Day Out London UK; ^4^ East London Community Centre London UK; ^5^ Antenatal Results and Choices London UK; ^6^ Alström Syndrome UK Torquay UK

## Abstract

**Objectives:**

Black and South Asian women in the UK face disproportionately worse pregnancy and maternal outcomes. Yet, they are underrepresented in research. Understanding their attitudes towards prenatal tests (screening tests and diagnostic genetic tests) is critical for offering equitable prenatal care.

**Methods:**

Focus groups examined attitudes towards prenatal testing amongst Black and South Asian parents. Discussions were analysed using reflexive thematic analysis.

**Results:**

Twelve Black and 15 South Asian parents participated in four focus groups. Four themes were identified: ‘The desire for information’, ‘The circle of trust’, ‘Faith and culture as navigators’, and ‘Knowledge and understanding of genetics’. Black and South Asian parents were open to prenatal screening tests, valuing the information about their baby's health. However, most opposed invasive testing because of the risks of harm to the baby. Wanting to be prepared, trust in healthcare, family influence and understanding of genetics shaped attitudes. Faith played a significant and varied role, with Muslim and Christian beliefs influencing decision‐making.

**Conclusion:**

This study underscores the need for culturally respectful prenatal care and the importance of building trust between healthcare services and Black and South Asian communities. It also highlights the value of including people from underrepresented populations in research for supporting health equity.

**Summary**

- What is already known about this topic?
  ◦ Examining parental attitudes towards prenatal testing helps our understanding of how to support parental decision‐making.
  ◦ Research is needed to understand how parents from communities underrepresented in research feel about prenatal testing.
- What does this study add?
  ◦ Parental attitudes are shaped by the desire for information, trust in healthcare, family influence, and religious and cultural beliefs.
  ◦ Culturally respectful counselling is needed, and tailored recruitment methods are essential for including people from diverse backgrounds in studies.

## 1 Introduction

Prenatal tests (screening tests and diagnostic genetic tests) are offered to identify the risk and presence of foetal anomalies during pregnancy. Whilst prenatal tests can provide parents with important information about their baby's health, and can provide clinically useful information for pregnancy, early neonatal management, and longer‐term prognosis [1, 2], there are emotional and psychological costs attached to receiving a prenatal diagnosis [3, 4]. These include considerations around termination [5] and the potential for receiving incidental findings that may have implications for the baby, parents, or wider family members [6].

Examining parental attitudes towards prenatal testing helps identify strategies to support families undergoing these tests. Insights from parents can also inform the development of effective pre‐ and post‐testing counselling. As technology advances and prenatal tests become more widely available, understanding these personal experiences is increasingly important.

A key issue, however, is that participants in many of the studies exploring views on prenatal testing do not reflect the demography of the wider population. Lacking diversity across various intersecting factors including ethnicity, education level and income, studies in this area have often represented the perspectives of women who are White, well‐educated, high‐earning or all three [7, 8, 9, 10]. Participants from different ethnic backgrounds have been included in maternal health studies across other contexts, but findings are sometimes aggregated into a single category [11, 12], which assumes homogeneity amongst people who have different values and cultural beliefs.

Studies examining views on newer genomic technologies continue to lack perspectives from people from non‐White backgrounds [10, 13, 14]. This is true even in our own work , where as part of an evaluation of the delivery of prenatal exome sequencing (pES) across England [15], we explored parental experiences of pES [16] but gathered limited insights from Black and South Asian parents. This underrepresentation impairs our understanding of the specific support needs of these communities, potentially leading to policies and guidelines that do not address their requirements.

The impact of this underrepresentation is compounded by existing inequalities in pregnancy and maternal outcomes, which see Black and South Asian women negatively and disproportionately impacted compared to women from White backgrounds [17, 18, 19]. Socioeconomic factors [20, 21, 22] and systemic racism [23, 24, 25] contribute to these disparities, influencing perceptions of care and engagement with maternity services [23, 26, 27]. As such, those facing the worst outcomes are those whose perspectives are least understood.

To address this cycle of exclusion and inequality, involving people from diverse backgrounds in studies is crucial. This is especially important when examining views on prenatal testing since religious principles and cultural beliefs may influence the acceptability of genetic services and understanding of genetic disease [28]. Broadening participation in this area could help ensure that prenatal testing services are equipped to offer equitable and culturally respectful care that aligns with parents' core beliefs and values.

Given this, and the fact that our own work with healthcare professionals has highlighted specific considerations on the acceptability of prenatal tests amongst parents from ethnic minority populations, we decided to ask parents from the two largest ethnic minority communities in the UK [29] for their views on prenatal tests directly. Guided by this work and studies elsewhere [19, 30, 31], the current study aimed to explore attitudes towards prenatal testing amongst Black and South Asian parents, with a focus on the role of religion, culture, and family and community networks. A better understanding of the factors that shape their attitudes is essential for guiding the development of equitable prenatal testing services.

## 2 Methods

### 2.1 Study Design

This qualitative study used focus groups with parents who identified as Black or South Asian. Focus groups were chosen to facilitate open discussion and enable the exploration of different viewpoints. In this work, we use the terms Black and South Asian as descriptors, as these are categories commonly used in the literature but fully acknowledge that people who identify as such come from many and varied communities within these high‐level categories.

### 2.2 Participants and Recruitment

Partnering with community leaders was crucial to this study; RBuabeng and LI are recognised within local Black and South Asian communities as trusted advocates. Electronic and print flyers about the study were distributed through RBuabeng and LI's connections, inviting potential participants to express interest by emailing the research team. Participants could take part if they or their partner were identified as Black (Black African, Black Caribbean, Black British or mixed Black heritage) or South Asian (South Asian, South Asian British or mixed South Asian heritage), were over 18, and had been pregnant within two years prior to the study. Of 32 who expressed interest, 27 participated. Prior to the focus group, all parents were given the option to provide demographic information that asked them to state their ethnicity, age, gender, and religious affiliation. Parents were also asked if they had had prior invasive prenatal genetic testing. Throughout this work, we refer to parents who self‐identified as Black African, Black Caribbean and Mixed Black Caribbean/White as Black (*n* = 12), and parents who self‐identified as Bangladeshi, Indian, and Pakistani (*n* = 15) as South Asian. Parents received a £40 gift voucher as a token of appreciation.

### 2.3 Focus Groups

Four focus groups were held between October and November 2023: two for Black parents and two for South Asian parents, with one online and one in‐person for each group. Sessions were separate to respect differences between the Black and South Asian communities and participants gave informed consent prior to the session. MP led all sessions, with RBuabeng and LI co‐facilitating the Black and South Asian groups, respectively. The in‐person session for South Asian parents was conducted in English and Bangla with LI interpreting. Development of the topic guide was guided by our previous research [16] and with input from members of parent support organisations (JF and KLB) who have years of experience of bringing a patient and public voice to research. RBuabeng and LI were also consulted to check that the questions were appropriate for the respective parent populations. Questions covered general views on prenatal screening tests and diagnostic genetic tests followed by open questions exploring the role of religion, culture, and family and community networks regarding decision‐making on these tests.

### 2.4 Data Analysis

Focus groups were digitally recorded, transcribed verbatim and pseudo‐anonymised before analysis. The focus group held in Bangla was transcribed verbatim into English and pseudo‐anonymised by LI. The focus groups were treated as a single dataset and analysed by MP (the sole coder) using reflexive thematic analysis (RTA)–an approach that allowed MP to be reflexive and engaged, respecting the subjective nature of parents' accounts [32], whilst exploring comparisons within and between parent groups. Immediate post‐focus group debriefs were held between MP, LI and RBuabeng during which key points and notable patterns were discussed, providing an opportunity to reflect on the data collaboratively and ensure that key insights were accurately. The analysis was predominantly inductive, involving open coding that centred on interpretative engagement with the data, with some deductive elements to ensure themes addressed the research questions [33]. The iterative process included data familiarisation, code generation, theme development, and theme review until a final thematic framework was established. The notion of coding reliability is philosophically misaligned to RTA [34]. As such, the findings were discussed with RBaptiste, RBuabeng and LI who acted not to validate data interpretation but as ‘critical friends’ to encourage consideration of alternative explanations [35]. These conversations served as a form of triangulation, helping to enhance the robustness of the analysis whilst respecting the reflexive nature of RTA. NVivo version 14 (QSR International, Pty Ltd) facilitated the analysis.

### 2.5 Reflexivity and Positionality

A central tenet of RTA is acknowledging the influence of the researcher's background on data interpretation. MP is Black British and has personal experience of prenatal testing. RTA embraces researcher subjectivity, and it was necessary to the analytic process that the data was interpreted through the lens of these experiences. Viewed and treated by society as an ‘ethnic minority person’ and being raised within a religious community–experiences that align with people from both groups, MP's shared cultural identity with those in the study afforded a nuanced understanding of the discussions and an enhanced relatability to the data [36].

## 3 Results

### 3.1 Participant Characteristics

Twenty‐seven parents attended four focus groups (online: *n* = 14; in‐person: *n* = 13). All parents were female, 55% were of South Asian heritage, and 45% were of Black and mixed Black heritage. Just under half (48%) spoke English as a second language, and 59% and 41% were of Muslim and Christian faiths, respectively. Six (22%) had had invasive prenatal testing in a previous pregnancy (Table 1).

**TABLE 1:** Participant characteristics.

	*N* (%)
Gender	
Female	27 (100)
Ethnicity	
Bangladeshi	13 (48)
Indian	1 (4)
Pakistani	1 (4)
Black African	6 (22)
Black Caribbean	5 (19)
Mixed black Caribbean/White	1 (4)
Education	
Degree or above	14 (52)
College/A‐level	5 (19)
Vocational	2 (7)
GCSE	2 (7)
Primary school	1 (4)
Unknown	3 (11)
Main language	
Bangla	4 (15)
Bangla‐Sylheti	8 (30)
English	13 (48)
Gujrati	1 (4)
Urdu	1 (4)
Religion	
Muslim	16 (59)
Christian	11 (41)
Age (years)	
25–34	8 (38)
35–44	13 (62)
Prior invasive testing	
Yes	6 (22)
No	21 (68)

### 3.2 Findings

**FIGURE 1:**
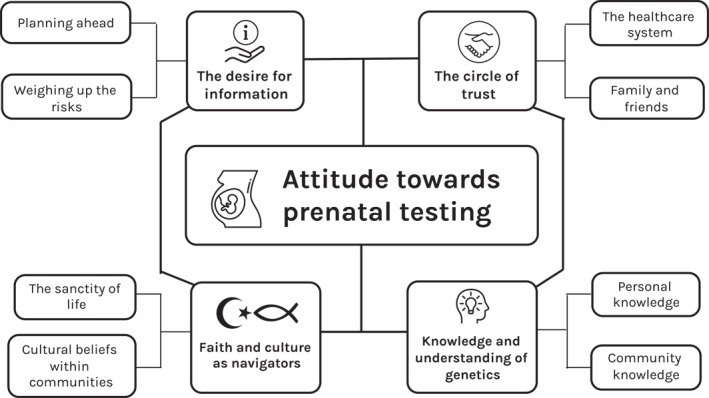
Thematic framework depicting the four constructs that shape attitudes towards prenatal testing.

Perspectives on prenatal testing fitted within a framework centred around one core concept: an individual's attitude towards prenatal testing. This attitude was shaped by four distinct but interrelated constructs, which mapped onto four overarching themes derived from the data: ‘*The desire for information*,’ ‘*The circle of trust*,’ ‘*Faith and culture as navigators*,’ and ‘*Knowledge and understanding of genetics*’ (Figure 1). Each theme is underpinned by the concept of ‘Belief,’ which varied in strength amongst individuals and influenced their behaviour in different ways: belief in the importance of preparedness could drive desire for knowledge about the baby's health; belief about who is a reliable, trustworthy source could determine the degree to which their input is sought or valued; belief guided by religious and cultural values could provide moral directives regarding whether prenatal testing aligns with personal or spiritual principles; and belief in one's understanding of genetics could influence personal perspectives on the necessity and reliability of prenatal testing.

#### 3.2.1 The Desire for Information

##### 3.2.1.1 Planning Ahead: ‘It Would Make Me More Prepared’

Both Black and South Asian parents expressed strong support for prenatal testing, viewing it as a valuable opportunity to gather information about their unborn baby's health. One parent summarised a common sentiment:

Genetic testing isn’t a bad thing…it’s finding out if your baby’s got a life limiting illness…it’s about…you know, I want to have more knowledge.

For most parents, the desire for information about their baby guided their behaviour; most had accepted the prenatal screening tests offered to all expectant parents through England's National Health Service (NHS). One parent stated, ‘I'd opt to be tested for everything,’ while another shared, ‘I've had the Down Syndrome test three times.’ Parents described wanting to be better prepared and avoid surprises after birth:

I think some of the tests I would go through with it… just to prepare myself so I know what I'm getting myself into.

Identifying an anomaly prenatally was also seen as helpful for allowing time to become familiar with the condition. Having this information could give parents the opportunity to seek potential treatments.

##### 3.2.1.2 Weighing up the risks: ‘I don't think I would put myself in that line of fire’

Importantly, the desire for more information did not mean that all prenatal tests were acceptable. There was a clear distinction between the acceptability of prenatal screening tests involving a blood test or ultrasound scan which were viewed as ‘just a normal test’, and diagnostic tests that used invasive methods as many parents were concerned about the risk of miscarriage associated with invasive procedures. The tension between the desire for information and putting the pregnancy at risk was highlighted by one parent who described the decision to undergo an invasive test as ‘an incredibly hard thing to wrestle with.’ Another echoed this sentiment, saying, ‘it makes me more confused. I want to do the test but when I heard the miscarriage chances, it puts me back, you know?’.

Only a few parents had opted for invasive testing. One Black parent, who received a prenatal diagnosis of Edward's syndrome, reflected positively on her decision:

Having that early testing for me was good… it was like monitoring and managing my own personal health as well, my own mental health.

However, the majority were strongly opposed, feeling that the associated risks outweighed the benefits. Notably, all seven Black parents in the online session indicated they would not consider invasive testing, as demonstrated by a parent who had been offered amniocentesis:

That’s what put me off doing it because of the risk of losing a child… I refused, from that time I refused.

Similar concerns were voiced by others who felt that undergoing the procedure ‘doesn't make sense’ and creates ‘fear.’ Another agreed, saying, ‘You wouldn't want to lose a baby at any stage, and you wouldn't want that because you were looking for a definitive answer.’

### 3.3 The Circle of Trust

#### 3.3.1 The Healthcare System: ‘I Don't Think I Was Fully Informed About It’

A significant factor influencing parents' attitudes towards prenatal testing was the level of trust in the healthcare system. Negative experiences with healthcare professionals (HCPs) often shape attitudes. Some experiences related specifically to prenatal testing; one parent criticised the pre‐test counselling she had received for invasive prenatal testing as ‘a very short discussion,’ whilst another recounted feeling distressed due to persistent enquiries from HCPs about termination:

I felt a lot of pressure from the medical field as well because they kept offering me the chance to abort my son, even after I had asked them to note it down that…I didn’t want to be asked anymore.

Others recounted negative experiences during pregnancy more generally which had eroded trust. Some felt ‘forced’ into procedures and not ‘fully informed’. Many of the Black parents contextualised their experiences within the framework of racial bias, expressing concerns about being perceived as ‘a problem patient’ and feeling they were in ‘protective mode all the time.’ One parent expressed anxiety that her pain would be dismissed:

They scanned and said that it was a twin pregnancy but one of the twins wasn’t developing properly…my biggest fear was that I would be in immense pain, and they would just see me as a Black woman in pain and not anything else.

South Asian parents were predominantly worried about the reliability of the prenatal tests themselves, citing instances where results suggested foetal anomalies but babies were born unaffected. Such experiences led to scepticism:

They told [her] there’s loads of problems in the pregnancy…when she’s born there is no problem…Alhamdulillah, she is now normal girl. So that’s what happens…sometimes it’s wrong, yeah, so that’s why I'm scared about those tests…that’s why I didn’t agree.

Notably, concerns around the potential misuse of personal genetic information were raised only by Black parents. One parent questioned the intentions of HCPs, stating, ‘I'm a bit sceptical about doing it [prenatal testing] because I don't trust them’, whilst another cited the Tuskegee syphilis study as an example of the scientific exploitation experienced by Black people, stating, ‘we only have to look in history to see what has been done.’

#### 3.3.2 Family and Friends: My Grandma Said I’m Going to be Used as a Guineapig… That’s Why I Chose Not to

Family and friends were held in high regard, and trust in their opinions and experiences could shape parents' attitudes and subsequent behaviour towards prenatal testing. Generational attitudes could play a significant role in decision‐making:

I think it [attitudes towards prenatal testing] is from the elders in the community…I was born in England, but I still had that thing where I just went with information that was provided.

These experiences were reiterated by a Black parent who had been advised against amniocentesis by her stepmother, and by a South Asian parent whose family member's advice had changed her perspective on prenatal screening tests in subsequent pregnancies:

She told me ‘Why did you do that test?’…next time you will remember because we don’t do the test because we believe in our God’…I trust her, so in my second pregnancy I didn’t do the Down Syndrome test because I know she’s telling something for my good.

### 3.4 Faith and Culture as Navigators

#### 3.4.1 The Sanctity of Life: ‘Every Child Is a Blessing’

Parents' attitudes towards prenatal screening and diagnostic testing were significantly influenced by their positionality on the sanctity of life and belief in the divine will. All parents identified as either Christian or Muslim, and their religious beliefs clearly shaped their perspectives on these tests. Amongst the Black parents, of whom 92% were Christian, a ‘pro‐life’ stance was prevalent; they viewed their role as being ‘a good steward of the pregnancy, even if they won't come to full term’. For them, discovering a genetic condition was challenging but did not necessarily alter their commitment to continuing the pregnancy. For many South Asian parents, religion acted as a direction with little room for negotiation. Many adhered to Islamic principles, holding strong views regarding termination or any procedure that could jeopardise their baby's life. One parent articulated this perspective:

Religiously it’s just not right…I think everyone that’s Muslim mostly generally thinks that in whatever situation, you should keep the baby.

Another shared that she had declined prenatal screening tests in all three pregnancies, ‘Because we are Muslim, and we trust in Allah.’

In other cases, parents utilised religion as guidance rather than instruction. Several Black parents balanced their faith with practical considerations, with many expressing the sentiment, ‘we believe in God for healing, but it's also good to know.’ Similarly, some South Asian parents acknowledged the nuances within their religious teachings. One parent felt that ‘there is a lot of misinformation that probably needs to be shared with Muslim women.’ Another described how consulting with her Imam after receiving a lethal prenatal diagnosis clarified that termination was permissible in her situation, aligning with her personal wishes.

#### 3.4.2 Cultural Beliefs Within Communities: ‘I Just Didn't Want That Judgement’

Cultural beliefs amongst family members and the wider communities did not always align with parents' needs, which could encourage covert behaviour. Some had undergone invasive procedures without telling family members, attending appointments alone and with little emotional support:

I told no‐one, except for one friend…I knew that my family would be really angry, and I knew their opinion on testing.

Stigma attached to invasive prenatal testing meant that some were reluctant to engage in conversations with family members, as with one parent who avoided the discussion ‘in case they thought I was pro‐abortion’. Disability was described as a source of shame amongst both Black and South Asian communities, further encouraging parents to conceal significant information about their baby's health:

One of my friends–she had a test done for Down Syndrome…it did come up positive…her husband wasn’t aware…she didn’t disclose that to any of his family, I think because of the stigma attached to the whole thing.

### 3.5 Knowledge and Understanding of Genetics

#### 3.5.1 Personal Knowledge: ‘I'm not Going to Lie; I Didn't Think It Happens to Black People’

Parents' understanding of genetics and genetic conditions significantly influenced their attitudes towards prenatal testing. Most understood genetics as ‘how parents transfer their genes and qualities through their children.’ While some parents accurately identified that some prenatal tests screen for conditions such as Down syndrome, others were less informed, believing that these tests could also detect conditions such as autism or cerebral palsy. Confusion about who genetic conditions affect was also evident; one South Asian parent described thalassaemia as ‘really a Black disease,’ and a Black parent had only recently learnt that Patau's syndrome could affect Black individuals.

#### 3.5.2 Community Knowledge: ‘It's Still a Very Big Taboo’

Both Black and South Asian parents noted a lack of understanding about prenatal testing and genetic conditions within their communities. This knowledge gap was sometimes attributed to lower education levels but could also be viewed as a cultural or generational issue as articulated by one parent:

I know if you told me the same information that you told my aunt who is probably ten years older than me…she would be much more reluctant to do any sort of testing.

Stigma and shame around disability were seen as direct consequences of these knowledge gaps about genetics, contributing to narratives where individuals, especially women, are perceived as failing to meet gendered social expectations. Older generations especially were found to blame individuals for genetic conditions, leading to reluctance in sharing health information for fear of ostracism.

Despite finding it ‘very difficult,’ parents acknowledged the benefits of sharing genetic history; they noted that the younger generation's openness to sharing experiences on social media is helping to raise awareness about genetics within communities. In some Black communities, pre‐marital genetic testing, often via the Church, is common, with these practices opening discussions about family inheritance and genetics:

They make us do a genetic test–a sickle cell test to determine if there’s any complications…it was a good conversation starter, and the Church won't allow you to get married until you do all of that.

## 4 Discussion

In this study, attitudes towards prenatal testing amongst Black and South Asian parents were shaped by key factors including personal desire for information, trust in key figures, cultural and religious values, and access to knowledge.

Parents from both Black and South Asian communities overwhelmingly supported prenatal screening tests. As others have found, these tests were seen as a valuable tool for understanding their baby's health and enabling preparation for the future [37, 38]. Notably, decisions to undergo screening tests were straightforward, with many viewing it as a simple blood test. However, it is possible that because these tests are positioned as safe and simple, pre‐test discussions about prenatal screening tests are not given the same weight as invasive tests by healthcare professionals. A potential risk is that parents may not afford prenatal screening tests the same level of consideration as they would an invasive procedure and so may be inadequately prepared for receiving a result that indicates a high chance of a foetal anomaly. Healthcare professionals should provide balanced pre‐test counselling that is balanced, and that supports parents' understanding of both the benefits and psychological implications of prenatal screening tests.

In comparison, almost all parents opposed prenatal tests that used invasive methods. These attitudes were heavily influenced by concerns about risks to the pregnancy as well as deeply held religious and cultural beliefs in a primarily Muslim and Christian cohort. This raises important questions about the type of support available to parents for whom a foetal anomaly is suspected but who decline confirmatory invasive testing. Future research should address this issue by examining how parents from Black and South Asian communities view procedures such as non‐invasive prenatal diagnosis (NIPD) which provide a diagnostic result without risk of harm to the baby [39]. Doing so could advance our understanding of how to support parents in making these decisions.

Religious and cultural beliefs exerted varying degrees of influence on individuals in this study: Black parents, most of whom identified as Christians, described faith as a source of emotional support during challenging decisions. Religious beliefs did not typically override medical advice; instead, faith was used as a coping mechanism, helping them balance their beliefs with their reality. In contrast, for most South Asian parents in this study, all of whom identified as Muslims, religious beliefs were central to their decision‐making, as has been reported elsewhere [40, 41]. Like other work [42], we uncovered important nuances in how religious beliefs are interpreted. It is critical to avoid making assumptions based on religious affiliation since levels of adherence and interpretation can vary within any faith group. HCPs should be mindful that the acceptability of termination in Islamic law is highly context‐dependent and that some parents may experience conflict or uncertainty about what their faith permits. In such cases, HCPs should be open to supporting parents in seeking religious guidance [43, 44] and be prepared to partner with religious scholars where appropriate.

Our study found that trust in healthcare systems significantly shaped attitudes towards prenatal testing. As in other studies, Black parents often expressed mistrust [45, 46, 47], rooted in historical mistreatment [48, 49, 50] and personal experiences of discrimination, echoing findings that negative healthcare encounters exacerbate health inequalities in global majority communities [51, 52]. To counteract this, HCPs must focus on developing trustworthiness and strengthening relationships with parents from these communities [53, 54]. Exposure to different cultures and undergoing training grounded in lived experiences is essential for supporting HCPs to recognise and respond appropriately to parents' cultural differences [55, 56].

Family and community perspectives were significant in shaping the attitudes of parents in this study. Misconceptions about prenatal testing and stigma around genetic conditions and disabilities are common in some cultures [57]. Some parents had hidden their testing decisions from their family for these reasons and for fear of marginalisation. By understanding the broader context in which attitudes are formed, HCPs can help parents feel supported and encourage sustained engagement with healthcare services. Fostering open dialogue about genetics [58] and normalising discussions on prenatal testing within local communities could support parents in making informed testing decisions.

## 5 Strengths and Limitations

Key to the success of this study was the involvement of community advocates, whose participation boosted interest in the study and facilitated insights into analysis. In addition, ethno‐cultural concordance between the facilitators and parents helped create a safe environment for parents to share their views. Further strengths included: the offering of an in‐person session which provided an intimate space for open discussion and accommodated children; the option of an online session for those unable to travel; a session conducted in Bangla to enable parents to engage in their native language; and adequate compensation for all parents. A limitation of the study is that parents who chose to take part may have done so because they had particularly strong views about the topic; thus, responder bias may be an issue. Furthermore, this study included 27 participants, all of whom were female and thus does not constitute a statistically representative sample of parents.

## 6 Conclusion

This study highlights the importance of implementing culturally respectful care for supporting informed decision‐making. HCPs should acknowledge the influence of cultural and religious beliefs when discussing prenatal testing but avoid making biased assumptions based on religious affiliation and ethnicity. Including these underrepresented groups in research is valuable, and tailored recruitment methods are important. A direct outcome of our approach has been the sustained relationship with the community of South Asian parents in this study. MP and JF (Director of parent charity Antenatal Results and Choices) hold regular sessions providing education about prenatal testing to parents from this community. This is a clear demonstration of how inclusive practices can lead to tailored support. By adopting more inclusive research and care approaches, healthcare services can better provide equitable support to families navigating prenatal testing decisions.

## Ethics Statement

Ethical approval for this study was given by the Health Research Authority (HRA) and the East of Scotland Research Ethics Service (EoSRES) on 14 August 2023 (REC Reference: 21/ES/0073).

## Consent

All participants were over the age of 18. Written informed consent was obtained from all participants to take part in this study and for this information to be published.

## Conflicts of Interest

The authors declare no conflicts of interest.

## Data Availability

The raw qualitative data that support the findings of this study contain sensitive information; therefore, we are unable to share the full transcripts from the focus groups. However, we may provide a summary of the discussions to researchers upon reasonable request from the corresponding author (MP) and where participant consent was given.

## References

[1] N. Chandler , S. Best , J. Hayward , et al., “Rapid Prenatal Diagnosis Using Targeted Exome Sequencing: A Cohort Study to Assess Feasibility and Potential Impact on Prenatal Counseling and Pregnancy Management,” Genetics in Medicine 20, no. 11 (2018): 1430–1437, 10.1038/gim.2018.30. 29595812

[2] L. K. Tolusso , P. Hazelton , B. Wong , and D. T. Swarr , “Beyond Diagnostic Yield: Prenatal Exome Sequencing Results in Maternal, Neonatal, and Familial Clinical Management Changes,” Genetics in Medicine 23, no. 5 (2021): 909–917, 10.1038/s41436-020-01067-9. 33442022 PMC7804210

[3] C. L. Hickerton , M. Aitken , J. Hodgson , and M. B. Delatycki , “Did You Find That Out in Time?": New Life Trajectories of Parents Who Choose to Continue a Pregnancy Where a Genetic Disorder Is Diagnosed or Likely,” American Journal of Medical Genetics 158a, no. 2 (2012): 373–383, 10.1002/ajmg.a.34399. 22140089

[4] A. Werner‐Lin , F. K. Barg , K. S. Kellom , et al., “Couple's Narratives of Communion and Isolation Following Abnormal Prenatal Microarray Testing Results,” Qualitative Health Research 26, no. 14 (2016): 1975–1987, 10.1177/1049732315603367. 26351292

[5] M. E. Norton , S. Nakagawa , and M. Kuppermann , “Women’s Attitudes Regarding Prenatal Testing for a Range of Congenital Disorders of Varying Severity,” Journal of Clinical Medicine 3, no. 1 (2014): 144–152: [Internet], 10.3390/jcm3010144. 26237253 PMC4449668

[6] L. Basel‐Salmon and R. Sukenik‐Halevy , “Challenges in Variant Interpretation in Prenatal Exome Sequencing,” European Journal of Medical Genetics 65, no. 2 (2022): 104410, 10.1016/j.ejmg.2021.104410. 34952236

[7] E. Hurford , A. Hawkins , L. Hudgins , and J. Taylor , “The Decision to Continue a Pregnancy Affected by Down Syndrome: Timing of Decision and Satisfaction With Receiving a Prenatal Diagnosis,” Journal of Genetic Counseling 22, no. 5 (2013): 587–593, 10.1007/s10897-013-9590-6. 23604903

[8] E. Quinlan‐Jones , S. C. Hillman , M. D. Kilby , and S. M. Greenfield , “Parental Experiences of Prenatal Whole Exome Sequencing (WES) in Cases of Ultrasound Diagnosed Fetal Structural Anomaly,” Prenatal Diagnosis 37, no. 12 (2017): 1225–1231, 10.1002/pd.5172. 29049852

[9] S. A. Walser , A. Werner‐Lin , A. Russell , R. J. Wapner , and B. A. Bernhardt , “Something Extra on Chromosome 5": Parents' Understanding of Positive Prenatal Chromosomal Microarray Analysis (CMA) Results,” Journal of Genetic Counseling 25, no. 5 (2016): 1116–1126. 26940446 10.1007/s10897-016-9943-z PMC5011030

[10] R. Mellis , D. Tapon , N. Shannon , et al., “Implementing a Rapid Fetal Exome Sequencing Service: What Do Parents and Health Professionals Think?,” Prenatal Diagnosis 42, no. 6 (2022): 783, 10.1002/pd.6140. 35383981 PMC9324936

[11] J. Henderson , H. Gao , and M. Redshaw , “Experiencing Maternity Care: The Care Received and Perceptions of Women From Different Ethnic Groups,” BMC Pregnancy and Childbirth 13, no. 1 (2013): 196, 10.1186/1471-2393-13-196. 24148317 PMC3854085

[12] J. Henderson and M. Redshaw , “Sociodemographic Differences in Women's Experience of Early Labour Care: A Mixed Methods Study,” BMJ Open 7, no. 7 (2017): e016351, 10.1136/bmjopen-2017-016351. PMC5541638 28710223

[13] J. Hammond , J. E. Klapwijk , S. Riedijk , et al., “Assessing Women's Preferences Towards Tests That May Reveal Uncertain Results From Prenatal Genomic Testing: Development of Attributes for a Discrete Choice Experiment, Using a Mixed‐Methods Design,” PLoS One 17, no. 1 (2022): e0261898, 10.1371/journal.pone.0261898. 35089945 PMC8797177

[14] C. G. Kernie , J. Wynn , A. Rosenbaum , et al., “Information Is Power: The Experiences, Attitudes and Needs of Individuals Who Chose to Have Prenatal Genomic Sequencing for Fetal Anomalies,” Prenatal Diagnosis 42, no. 7 (2022): 947–954, 10.1002/pd.6153. 35476893 PMC11561471

[15] M. Hill , S. Ellard , J. Fisher , et al., “Optimising Exome Prenatal Sequencing Services (EXPRESS): A Study Protocol to Evaluate Rapid Prenatal Exome Sequencing in the NHS Genomic Medicine Service,” NIHR Open Res 2 (2022): 10, 10.3310/nihropenres.13247.2. 35935673 PMC7613246

[16] H. McInnes‐Dean , R. Mellis , M. Daniel , et al., “'Something That Helped the Whole Picture': Experiences of Parents Offered Rapid Prenatal Exome Sequencing in Routine Clinical Care in the English National Health Service,” Prenatal Diagnosis 44, no. 4 (2024): 465–479, 10.1002/pd.6537. 38441167

[17] Centers for Disease Control and Prevention , “Pregnancy Mortality Surveillance System.” 2024, Contract No.: 06/09/2024.

[18] M. B. K. Knight , A. Felker , R. Patel , R. Kotnis , S. Kenyon , and J. J. Kurinczuk , eds., Saving Lives, Improving Mothers’ Care Core Report ‐ Lessons Learned to Inform Maternity Care from the UK and Ireland Confidential Enquiries into Maternal Deaths and Morbidity 2019‐21 (National Perinatal Epidemiology Unit, University of Oxford, 2023).

[19] Webster K. N. P. T. “Ethnic and Socio‐Economic Inequalities in NHS Maternity and Perinatal Care for Women and Their Babies: Assessing Care Using Data From Births Between 1 April 2015 and 31 March 2018 Across England, Scotland and Wales;” 2021.

[20] Y. Kelly , L. Panico , M. Bartley , M. Marmot , J. Nazroo , and A. Sacker , “Why Does Birthweight Vary Among Ethnic Groups in the UK? Findings From the Millennium Cohort Study,” Journal of Public Health 31, no. 1 (2009): 131–137, 10.1093/pubmed/fdn057. 18647751

[21] H. H. Burris and M. R. Hacker , “Birth Outcome Racial Disparities: A Result of Intersecting Social and Environmental Factors,” Seminars in Perinatology 41, no. 6 (2017): 360–366, 10.1053/j.semperi.2017.07.002. 28818300 PMC5657505

[22] N. Vousden , K. Bunch , S. Kenyon , J. J. Kurinczuk , and M. Knight , “Impact of Maternal Risk Factors on Ethnic Disparities in Maternal Mortality: A National Population‐Based Cohort Study,” Lancet Reg Health Eur 40 (2024): 100893, 10.1016/j.lanepe.2024.100893. 38585675 PMC10998184

[23] V. S. Raleigh , D. Hussey , I. Seccombe , and K. Hallt , “Ethnic and Social Inequalities in Women's Experience of Maternity Care in England: Results of a National Survey,” Journal of the Royal Society of Medicine 103, no. 5 (2010): 188–198, 10.1258/jrsm.2010.090460. 20436027 PMC2862068

[24] J. Jomeen and M. Redshaw , “Ethnic Minority Women's Experience of Maternity Services in England,” Ethnicity and Health 18, no. 3 (2013): 280–296, 10.1080/13557858.2012.730608. 23039872

[25] Peter M. W. R. “The Black Maternity Experiences Report: A Nationwide Study of Black Women’s Experiences of Maternity Services in the United Kingdom.” 2022 May 2022.

[26] G. Thomson , J. Cook , N. Crossland , et al., “Minoritised Ethnic Women's Experiences of Inequities and Discrimination in Maternity Services in North‐West England: A Mixed‐Methods Study,” BMC Pregnancy and Childbirth 22, no. 1 (2022): 958, 10.1186/s12884-022-05279-6. 36550440 PMC9773462

[27] M. Knight , K. Bunch , N. Vousden , et al., “A National Cohort Study and Confidential Enquiry to Investigate Ethnic Disparities in Maternal Mortality,” EClinicalMedicine 43 (2022): 101237, 10.1016/j.eclinm.2021.101237. 34977514 PMC8683666

[28] A. Zhong , B. Darren , B. Loiseau , et al., “Ethical, Social, and Cultural Issues Related to Clinical Genetic Testing and Counseling in Low‐ and Middle‐Income Countries: A Systematic Review,” Genetics in Medicine 23, no. 12 (2021): 2270–2280, 10.1038/s41436-018-0090-9. 30072741

[29] GOV.UK , “Population of England and Wales,”2022, : [Available from:, https://www.ethnicity‐facts‐figures.service.gov.uk/uk‐population‐by‐ethnicity/national‐and‐regional‐populations/population‐of‐england‐and‐wales/latest/.

[30] S. Ahmed , K. Atkin , J. Hewison , and J. Green , “The Influence of Faith and Religion and the Role of Religious and Community Leaders in Prenatal Decisions for Sickle Cell Disorders and Thalassaemia Major,” Prenatal Diagnosis 26, no. 9 (2006): 801–809, 10.1002/pd.1507. 16927359

[31] K. Atkin , S. Ahmed , J. Hewison , and J. M. Green , “Decision‐Making and Ante‐natal Screening for Sickle Cell and Thalassaemia Disorders: To what Extent Do Faith and Religious Identity Mediate Choice?,” Current Sociology 56, no. 1 (2008): 77–98, 10.1177/0011392107084380.

[32] V. C. V. Braun , Thematic Analysis: A Practical Guide (Sage Publications Ltd, 2021).

[33] V. Braun and V. Clarke , “A Critical Review of the Reporting of Reflexive Thematic Analysis in Health Promotion International,” Health Promotion International 39, no. 3 (2024), 10.1093/heapro/daae049. PMC11132294 38805676

[34] V. Braun and V. Clarke , “Toward Good Practice in Thematic Analysis: Avoiding Common Problems and Be(com)ing a Knowing Researcher,” Int J Transgend Health 24, no. 1 (2023): 1–6, 10.1080/26895269.2022.2129597. 36713144 PMC9879167

[35] B. Smith and A. C. Sparkes , “Narrative Inquiry in Psychology: Exploring the Tensions within,” Qualitative Research in Psychology 3, no. 3 (2006): 169–192, 10.1191/1478088706qrp068oa.

[36] R. Berger , “Now I See it, Now I Don’t: Researcher’s Position and Reflexivity in Qualitative Research,” Qualitative Research 15, no. 2 (2013): 219–234, 10.1177/1468794112468475.

[37] E. Floyd , M. A. Allyse , and M. Michie , “Spanish‐ and English‐Speaking Pregnant Women's Views on cfDNA and Other Prenatal Screening: Practical and Ethical Reflections,” Journal of Genetic Counseling 25, no. 5 (2016): 965–977, 10.1007/s10897-015-9928-3. 26739840 PMC4936962

[38] C. Lewis , C. Silcock , and L. S. Chitty , “Non‐invasive Prenatal Testing for Down's Syndrome: Pregnant Women's Views and Likely Uptake,” Public Health Genomics 16, no. 5 (2013): 223–232, 10.1159/000353523. 23886854

[39] S. Drury , S. Mason , F. McKay , et al., “Implementing Non‐invasive Prenatal Diagnosis (NIPD) in a National Health Service Laboratory; From Dominant to Recessive Disorders,” Advances in Experimental Medicine and Biology 924 (2016): 71–75, 10.1007/978-3-319-42044-8_14. 27753022

[40] A. Njenga , “Somali Refugee Women's Experiences and Perceptions of Western Health Care,” Journal of Transcultural Nursing 34, no. 1 (2023): 8–13, 10.1177/10436596221125893. 36197048

[41] S. M. Hassan , C. Leavey , and J. S. Rooney , “Exploring English Speaking Muslim Women's First‐Time Maternity Experiences: A Qualitative Longitudinal Interview Study,” BMC Pregnancy and Childbirth 19, no. 1 (2019): 156, 10.1186/s12884-019-2302-y. 31060520 PMC6501380

[42] J. C. Lewis Ch , M. Riddington , M. Hill , et al., “Minimally Invasive Autopsy for Fetuses and Children Based on a Combination of Post‐mortem MRI and Endoscopic Examination: A Feasibility Study,” Health Technology Assessment 23, no. 46 (2019): 1–104, 10.3310/hta23460. PMC6732714 31461397

[43] S. A. Eyerly‐Webb , S. Jumale , I. Wolfe , et al., “Considerations for Specialized Maternal‐Fetal Care in the Somali‐American Community,” Prenatal Diagnosis (2024). 10.1002/pd.6625 38991746

[44] A. I. Padela , A. Killawi , M. Heisler , S. Demonner , and M. D. Fetters , “The Role of Imams in American Muslim Health: Perspectives of Muslim Community Leaders in Southeast Michigan,” Journal of Religion and Health 50, no. 2 (2011): 359–373, 10.1007/s10943-010-9428-6. 21088896

[45] E. J. Gordon , D. Amórtegui , I. Blancas , C. Wicklund , J. Friedewald , and R. R. Sharp , “A Focus Group Study on African American Living Donors' Treatment Preferences, Sociocultural Factors, and Health Beliefs About Apolipoprotein L1 Genetic Testing,” Progress in Transplantation 29, no. 3 (2019): 239–247. 31146624 10.1177/1526924819854485

[46] D. Edge , “It's Leaflet, Leaflet, Leaflet Then, "see You Later"': Black Caribbean Women's Perceptions of Perinatal Mental Health Care,” British Journal of General Practice 61, no. 585 (2011): 256–262, 10.3399/bjgp11x567063. PMC3063015 21439184

[47] O. Ojo‐Aromokudu , A. Suffel , S. Bell , and S. Mounier‐Jack , “Views and Experiences of Primary Care Among Black Communities in the United Kingdom: A Qualitative Systematic Review,” Ethnicity and Health 28, no. 7 (2023): 1006–1025, 10.1080/13557858.2023.2208313. 37160684

[48] R. V. Katz , S. S. Kegeles , N. R. Kressin , et al., “Awareness of the Tuskegee Syphilis Study and the US Presidential Apology and Their Influence on Minority Participation in Biomedical Research,” American Journal of Public Health 98, no. 6 (2008): 1137–1142, 10.2105/ajph.2006.100131. 17901437 PMC2377291

[49] J. M. Sims , The Story of My Life H. Sims JMMS , ed. (Joseph Meredith Toner Collection, 1894).

[50] S. O. Sodeke and L. R. Powell , “Paying Tribute to Henrietta Lacks at Tuskegee University and at the Virginia Henrietta Lacks Commission,” Journal of Health Care for the Poor and Underserved 30, no. 4s (2019): 1–11, 10.1353/hpu.2019.0109. PMC7484676 31735712

[51] A. E. Shields , W. Burke , and D. E. Levy , “Differential Use of Available Genetic Tests Among Primary Care Physicians in the United States: Results of a National Survey,” Genetics in Medicine 10, no. 6 (2008): 404–414, 10.1097/gim.0b013e3181770184. 18496223 PMC2764316

[52] N. Peters , A. Rose , and K. Armstrong , “The Association Between Race and Attitudes About Predictive Genetic Testing,” Cancer Epidemiology, Biomarkers & Prevention 13, no. 3 (2004): 361–365, 10.1158/1055-9965.361.13.3. 15006909

[53] S. M. Sharif , M. Blyth , M. Ahmed , et al., “Enhancing Inclusion of Diverse Populations in Genomics: A Competence Framework,” Journal of Genetic Counseling 29, no. 2 (2020): 282–292, 10.1002/jgc4.1263. 32250032

[54] W. J. Ferguson and L. M. Candib , “Culture, Language, and the Doctor‐Patient Relationship,” Family Medicine 34, no. 5 (2002): 353–361. 12038717

[55] S. Esegbona‐Adeigbe , “Acquiring Cultural Competency in Caring for Black African Women,” British Journal of Midwifery 19, no. 8 (2011): 489–496, 10.12968/bjom.2011.19.8.489.

[56] L. Goodwin , B. Hunter , and A. Jones , “The Midwife‐Woman Relationship in a South Wales Community: Experiences of Midwives and Migrant Pakistani Women in Early Pregnancy,” Health Expectations 21, no. 1 (2018): 347–357, 10.1111/hex.12629. 28960699 PMC5750740

[57] F. K. Boardman , C. Clark , E. Jungkurth , and P. J. Young , “Social and Cultural Influences on Genetic Screening Programme Acceptability: A Mixed‐Methods Study of the Views of Adults, Carriers, and Family Members Living With Thalassemia in the UK,” Journal of Genetic Counseling 29, no. 6 (2020): 1026–1040, 10.1002/jgc4.1231. 32114710 PMC7754126

[58] N. Khan , G. Kerr , and H. Kingston , “Community Engagement and Education: Addressing the Needs of South Asian Families With Genetic Disorders,” Journal of Community Genetics 7, no. 4 (2016): 317–323, 10.1007/s12687-016-0278-0. 27614444 PMC5138164

